# High mammographic density in women of Ashkenazi Jewish descent

**DOI:** 10.1186/bcr3424

**Published:** 2013-05-13

**Authors:** Jennifer L Caswell, Karla Kerlikowske, John A Shepherd, Steven R Cummings, Donglei Hu, Scott Huntsman, Elad Ziv

**Affiliations:** 1 Department of Medicine, Division of General Internal Medicine, Institute for Human Genetics, Helen Diller Family Comprehensive Cancer Center, University of California, 1450 3rd Street, San Francisco, CA 94143, USA; 2 Department of Medicine and Department of Epidemiology and Biostatistics, University of California, San Francisco, San Francisco Veterans Affairs Medical Center, General Internal Medicine Section 111A1, 4150 Clement Street, San Francisco, CA 94121, USA; 3 Department of Radiology and Biomedical Imaging, University of California, San Francisco, MRSC AC122, 1 Irving Street (Box 0628), San Francisco, CA 94143-0628, USA; 4 San Francisco Coordinating Center, California Pacific Medical Center Research Institute, 185 Berry St., Lobby 5, Suite 5700, San Francisco, CA, 94107, USA

## Abstract

**Introduction:**

Percent mammographic density (PMD) adjusted for age and body mass index is one of the strongest risk factors for breast cancer and is known to be approximately 60% heritable. Here we report a finding of an association between genetic ancestry and adjusted PMD.

**Methods:**

We selected self-identified Caucasian women in the California Pacific Medical Center Research Institute Cohort whose screening mammograms placed them in the top or bottom quintiles of age-adjusted and body mass index-adjusted PMD. Our final dataset included 474 women with the highest adjusted PMD and 469 with the lowest genotyped on the Illumina 1 M platform. Principal component analysis (PCA) and identity-by-descent analyses allowed us to infer the women's genetic ancestry and correlate it with adjusted PMD.

**Results:**

Women of Ashkenazi Jewish ancestry, as defined by the first principal component of PCA and identity-by-descent analyses, represented approximately 15% of the sample. Ashkenazi Jewish ancestry, defined by the first principal component of PCA, was associated with higher adjusted PMD (*P *= 0.004). Using multivariate regression to adjust for epidemiologic factors associated with PMD, including age at parity and use of postmenopausal hormone therapy, did not attenuate the association.

**Conclusions:**

Women of Ashkenazi Jewish ancestry, based on genetic analysis, are more likely to have high age-adjusted and body mass index-adjusted PMD. Ashkenazi Jews may have a unique set of genetic variants or environmental risk factors that increase mammographic density.

## Introduction

Percent mammographic density (PMD) is the proportion of radiographically dense breast tissue as a fraction of the entire breast and can be calculated from a two-dimensional mammogram image [1-3] or as a fraction of the entire volume of the breast [4-13]. PMD is a strong risk factor for breast cancer; women of the same age and body mass index (BMI) in the upper quartile of PMD have a fourfold to sixfold higher risk of breast cancer than women in the lower quartile [1,3,14-20].

Many of the risk factors for high PMD are also risk factors for breast cancer, including late parity and use of postmenopausal hormone therapy with estrogen and progestin [3,21]. However, reproductive and hormonal factors account for a small proportion of the variation in PMD [21], and PMD remains a risk factor for breast cancer when adjusting for these factors [22,23]. Approximately 60% of the variance in PMD is heritable [24-27] and some genetic variants that are associated with breast cancer risk are also associated with increased PMD [28]. Both linkage and genome-wide association studies have been used to search for genetic determinants of PMD [29-33]. To date, the majority of heritability remains unexplained; for example, a recent genome-wide association study found SNP variants accounting for only 0.5% of the variance in PMD [30].

Identifying an ethnic population with higher PMD may have implications for breast cancer risk in that population and could open new avenues to map genes for this trait. We genotyped US Caucasian women at the extremes of adjusted PMD and evaluated the association between genetic ancestry and adjusted PMD, uncovering a previously unknown association between Ashkenazi Jewish ancestry and adjusted PMD.

## Methods

### Study sample

Study subjects were selected from 4,511 women enrolled in the California Pacific Medical Center Breast Health Cohort who underwent screening mammography between January 2004 and April 2006 and consented to provide blood specimens between July 2004 and June 2007. The California Pacific Medical Center Breast Health Cohort is linked to the San Francisco Mammography Registry, part of the NCI Breast Cancer Surveillance Consortium that collects demographic and risk factor data on women receiving mammography.

The questionnaire includes information on age, race, height, weight, parity history, postmenopausal hormone therapy use, personal history of breast cancer, and family history of breast cancer (in mother, sister, or daughter). The questionnaire allows the following categories for race/ethnicity: White/Caucasian, Black/African American, Hispanic/Latina, American Indian, Chinese, Japanese, Filipina, Vietnamese, Other Asian and Other; it did not include Ashkenazi Jewish as a category. Only women who reported White/Caucasian race/ethnicity were included in this study. We excluded women who reported a personal history of breast cancer.

All participants gave informed consent to participate in the research. The study was approved by the University of California, San Francisco and the California Pacific Medical Center institutional review boards.

### Measurement of mammographic density

PMD was calculated from craniocaudal digitized film mammograms using single X-ray absorptiometry (SXA), as described in [4]. In brief, the method makes two separate calculations: the total volumetric density and the total breast density. PMD is calculated as the quotient of total volumetric density and total breast volume. To calculate the total volumetric density, a calibrated phantom reference material is placed in the unused corner of the film mammogram. The phantom is composed of two materials, one the same density as fat and the other the same density as fibroglandular tissue. For each pixel of the mammogram, the percent density is calculated based on where that pixel falls on the gray scale from the low-density material to the high-density material. Total volumetric density is then calculated as the average of these values across all breast pixels. Total breast volume is calculated based on the distance between the X-ray source and detector and an algorithm that takes into account the tilt of the source/detector and the shape of the compressed breast.

This method has been shown to be highly reproducible [4], and to be at least as strongly associated with breast cancer risk as traditionally estimated PMD [5]. We used the average PMD of the right and left breasts. For women who only had a value for PMD on one side, we used the measurement from the side with data.

### Selection of women with high and low adjusted percent mammographic density

We used age-adjusted and BMI-adjusted PMD to select participants for the genetic study. We square root-transformed PMD to make the data less skewed (Figure S1 in Additional File 1) and then used a linear regression model to calculate the association between age, BMI, and square root-transformed PMD. We used the residuals of this model as the adjusted PMD value for each woman. We selected 500 women with adjusted PMD in the highest quintile and 500 women with adjusted PMD in the lowest quintile for genotyping. Of these, we were able to identify 494 corresponding biospecimens from women in the top quintile and 489 biospecimens from women in the bottom quintile. All 1,000 women were linked to the California Cancer Registry by the San Francisco Mammography Registry annually since 2004 to confirm the women did not develop breast cancer after their screening examination.

### Genotyping

The samples were genotyped on the Illumina 1 M platform at the Center for Inherited Disease Research. A total of 40 samples were excluded from further analysis because they were unexpected duplicates of other samples (*n *= 7), they did not perform well in genotyping (*n *= 25), they did not cluster with European samples in principal component analysis (PCA) (*n *= 4) (Figure S2 in Additional File 1), they appeared in the dataset as both a high-density sample and a low-density sample (*n *= 1), they were found to be unexpected full siblings of other samples (*n *= 2), or they did not have an associated phenotype (*n *= 1) (Table S1 in Additional File 1), leaving 474 women with high adjusted PMD and 469 women with low adjusted PMD. Of the 1,043,142 SNPs genotyped, 45,933 were excluded from further analysis because they had no position information, they were mitochondrial or on the Y-chromosome, they were intensity-only or technical failures, or they had greater than 10% failed genotyping (Table S2 in Additional File 1).

### Statistical analyses

We used *t *tests and Wilcoxon rank-sum tests for continuous variables and used chi-square tests for categorical variables to determine whether there were significant differences between women with high adjusted PMD and women with low adjusted PMD for baseline characteristics and principal components (PCs).

**Figure 1:**
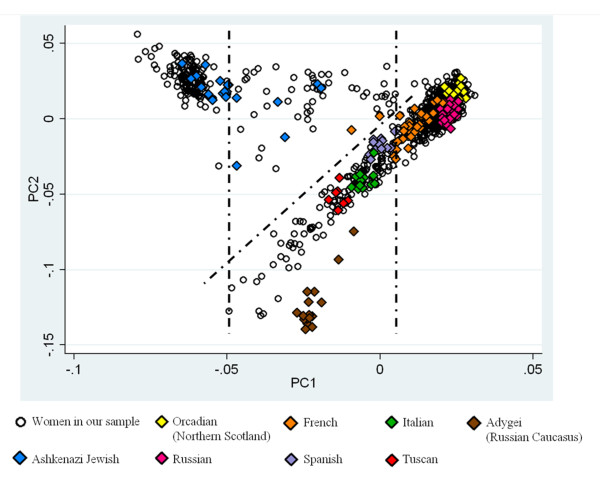
**Principal component analysis of participants from our study and additional reference samples of known ancestry**. The first principal component (PC1) separates people of Ashkenazi Jewish ancestry from other European groups. The vertical dotted lines separate the Ashkenazi Jewish and Northern European clusters from the middle group. The diagonal dotted line divides the probably mixed Ashkenazi-other European group (higher values on the second principal component (PC2)) from the Southern European group (lower values on PC2).

We performed PCA using EIGENSTRAT [34]. To infer ancestry, we included publicly available [35,36] genotype datasets from European and Middle Eastern samples with known ancestry: Italians (*n *= 14), Tuscans (*n *= 8), Basque (*n *= 24), French (*n *= 29), Orcadians (*n *= 16), Russians (*n *= 27), Adygei (*n *= 17), Sardinians (*n *= 28), Spanish (*n *= 12), Ashkenazi Jews (*n *= 21), and Palestinians (*n *= 51). For the combined PCA analysis, we used a subset of 390,144 SNPs that were genotyped on both the Illumina 1 M platform and the other Illumina platforms used in the reference datasets. Changing the European and Middle Eastern groups did not substantially change the first principal component (PC1) (data not shown). For visualization of results in Figure 1, we selected the ancestry groups that most closely corresponded in the PC space to the Caucasian American women of our sample; these groups included the Italians, Tuscans, French, Orcadians, Russians, Adygei, Spanish, and Ashkenazi Jews.

We performed analysis of shared extended haplotypes (identity by descent (IBD)) using GERMLINE [37]. We defined a shared extended haplotype as being at least three centimorgans long, the default setting for GERMLINE. Prior to running GERMLINE we phased the genotype data and imputed missing genotypes using BEAGLE [38]. We calculated 95% confidence intervals (CIs) of the mean shared IBD between groups, as well as *P *values comparing two different means, using bootstrapping, resampling 1,000 times.

We performed multivariate logistic regression analysis on the outcome of high versus low adjusted PMD to assess whether the association between position on PC1 and adjusted PMD remained significant after adjusting for baseline characteristics using Stata software (version 10.0; Stata Corporation, 4905 Lakeway Dr., College Station, TX, 77845). For this analysis, we performed a linear transformation on PC1 in order to quantify the percent Ashkenazi Jewish ancestry on a scale of approximately 0 to 1:

$$\left(\text{Value of PC1}-0.0\text{3}0\text{9}\right)/-0.\text{11}0\text{2}$$

After this transformation, a PC1 value of -0.0793 became 1.0, corresponding to the highest value of Ashkenazi Jewish ancestry in our sample, and a PC1 value of 0.1102 became 0.0 and was associated with the greatest distance along PC1 from individuals of Ashkenazi Jewish ancestry.

## Results

### Characteristics of women with high versus low adjusted percent mammographic density

The mammographic and epidemiologic characteristics of women with high versus low age-adjusted and BMI-adjusted PMD are presented in Table 1. Unadjusted PMD and volume of mammographic density were significantly higher and total breast volume was lower in women with high adjusted PMD. Postmenopausal women were more likely to have lower adjusted PMD. Women with high adjusted PMD were more likely to have reported a family history of breast cancer.

**Table 1:** Characteristics of women with high adjusted percent mammographic density versus low adjusted percent mammographic density

	High adjusted PMD	Low adjusted PMD	*P *value
Percent dense tissue by volume	72.7 (13.3)	29.6 (6.4)	<10^-3^
Volume of dense breast tissue (ml)	327.3 (215.0)	166.2 (70.2)	<10^-3^
Volume of the breast (ml)	479.1 (354.6)	576.5 (242.9)	<10^-3^
Age (years)	52.4 (9.7)	51.7 (7.8)	0.9
Body mass index (kg/m^2^)	24.1 (6.5)	23.9 (3.1)	<10^-3^
Age at first live birth			0.1
<30 years	96 (20%)	114 (24%)	
≥30 years or nulliparous	378 (80%)	354 (76%)	
Unknown	0	1	
Menopausal status			
Premenopausal	203 (43.3%)	159 (34.3%)	
Postmenopausal	157 (33.5%)	183 (39.4%)	0.009*
Postmenopausal on hormone therapy	71 (15.1%)	77 (16.6%)	0.7**
Unknown	38 (8.1%)	45 (9.7%)	
First-degree relative with breast cancer			0.02
Yes	114 (24%)	84 (18%)	
No	366 (76%)	383 (82%)	
Unknown	0	2	

Data presented as mean (standard deviation) or *n *(%). PMD, percent mammographic density. Chi-squared *P *values calculated excluding the samples with unknown values. *P value for comparison with premenopausal women. **P value for comparison with postmenopausal women not taking hormone therapy.

BMI was significantly higher in women with high adjusted PMD by the Wilcoxon rank-sum test, but not by the *t *test (*P *= 0.6). Our initial method of adjusting PMD for BMI assumed a linear relationship between BMI and PMD, while the relationship is in fact nonlinear, especially at higher BMI values (Figure S3 in Additional File 1). Women with higher BMI values were therefore over-represented in the group with higher adjusted PMD.

### Identification of population substructure

We performed PCA to determine the population substructure of our sample. First we performed PCA with populations of European, African, Asian, American, and Oceanian descent to verify the Caucasian ancestry of our sample population. Of the 951 women included in the initial analysis, four women appeared to have a possible admixture with Asian or African ancestry (Figure S2 in Additional File 1). To simplify the analysis, we excluded these women from additional analyses. Next, we performed the PCA again with only the population that clustered with European ancestry and incorporated publicly available genotyped samples from European and Middle Eastern populations of known ancestry (Figure 1). PC1 separated people of Ashkenazi Jewish ancestry from other European groups. Excluding the ancestral populations had no effect on PC1; the correlation of the component scores of PC1 when including versus excluding these samples of known ancestry was *r^2 ^*= 1.0. The second PC reflected Northern versus Southern European ancestry (Figure 1). When we excluded the ancestral populations, the component scores of the third PC were highly correlated with the component scores of the second PC with ancestral populations (*r^2 ^*= 0.88).

Ashkenazi Jews have a significantly higher proportion of their genome that is IBD than other Caucasian populations [39,40]. We therefore performed analysis of IBD in our sample. We first defined two clusters in the PC space: a probable Northern European cluster (Group 1) with PC1 ≥0.005 and a probable Ashkenazi Jewish cluster (Group 4) with PC1 ≤-0.0495 (Figure 1). Group 1 represented 65.7% of the total Caucasian sample and Group 4 represented 15.8%. We compared the degree of IBD among pairs of women within each of the groups. The pairs of individuals in Group 4 averaged 23.2 centimorgans of shared haplotypes compared with 6.0 centimorgans in the pairs from Group 1 (bootstrap *P *< 10^-3^), consistent with the hypothesis that Group 4 represented women of Ashkenazi Jewish descent.

### Ashkenazi Jewish ancestry is associated with high adjusted percent mammographic density

**Figure 2:**
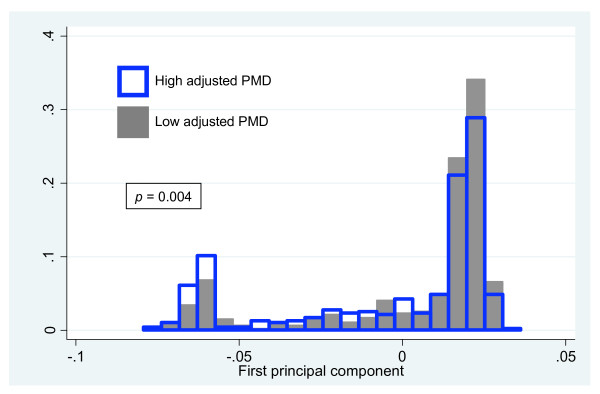
**Position along the first principal component correlates with risk of high/low adjusted percent mammographic density**. *P *value of association between the first principal component and percent mammographic density (PMD) obtained using the Wilcoxon rank-sum test.

We examined the association between PCs from the genetic ancestry analysis and adjusted PMD. We identified a significant association between PC1 and adjusted PMD (*P *= 0.004). Comparing the distributions of PC1 values between women with high and low adjusted PMD, we found that women with low PC1 values were over-represented in the high adjusted PMD group and women with high PC1 values were over-represented in the low adjusted PMD group (Figure 2). Using transformed values of PC1, with 0 representing the least amount of Ashkenazi Jewish ancestry as measured by PCA and 1 representing the greatest, having a PC1 value of 1 corresponded to an odds ratio (OR) of 2.0 for having high adjusted PMD. To adjust for the nonlinear relationship between BMI and PMD, we re-adjusted for BMI by quartiles (Table 2) and by deciles (Table S3 in Additional File 1) in multivariate analysis. The multivariate adjusted OR for the association of PC1 and adjusted PMD after adjusting for BMI by quartiles is 2.2 (95% CI = 1.4 to 3.6). The same OR after adjusting for BMI by deciles is 2.2 (95% CI = 1.3 to 3.6). We also analyzed our data by clustering women into subgroups based on the PCA results (Figure 1). The group of women that clustered with Ashkenazi Jews (Group 4) had an OR of 1.60 (*P *= 0.01; Table S4 in Additional File 1) of having high adjusted PMD compared with the group of women who clustered Northern Europeans (Group 1).

**Table 2:** Multivariate regression of baseline characteristics and the first principal component with adjusted percent mammographic density

	Univariate analyses		Multivariate analysis	
	
	Odds ratio (95% confidence interval)	*P *value	Odds ratio (95% confidence interval)	*P *value
First principal component	2.00 (1.26 to 3.17)	0.003	2.22 (1.36 to 3.65)	0.002
Body mass index				
First quartile (<21 kg/m^2^)	Reference		Reference	
Second quartile (21 to 23 kg/m^2^)	0.41 (0.28 to 0.59)	<0.001	0.38 (0.26 to 0.57)	<0.001
Third quartile (23 to 26 kg/m^2^)	0.33 (0.23 to 0.49)	<0.001	0.31 (0.21 to 0.46)	<0.001
Fourth quartile (>26 kg/m^2^)	0.44 (0.30 to 0.63)	<0.001	0.48 (0.32 to 0.71)	<0.001
Age at first live birth				
<30 years	Reference		Reference	
≥30 years or nulliparous	1.27 (0.93 to 1.72)	0.1	1.13 (0.80 to 1.59)	0.5
Menopausal status				
Premenopausal	Reference		Reference	
Postmenopausal	0.67 (0.50 to 0.90)	0.009	0.67 (0.49 to 0.92)	0.01
Postmenopausal on hormone therapy	0.72 (0.49 to 1.06)	0.1	0.74 (0.49 to 1.10)	0.1

Odds ratios are for likelihood of having high adjusted percent mammographic density compared with low adjusted percent mammographic density. Univariate and multivariate *P *values are calculated using chi-squared tests.

The seventh PC was also associated with adjusted PMD (*P *= 0.03), although we were unable to identify a correlation with an ancestral population and the seventh PC. The third PC (which corresponded to the second PC in the analysis with the ancestral populations and separated Northern versus Southern European ancestry) did not correlate with adjusted PMD (*P *= 0.3). Table S5 in Additional File 1 presents the correlation of each of the first 10 PCs with adjusted PMD.

Adjusting for the known epidemiologic and mammographic characteristics of the women in our sample did not attenuate the association between Ashkenazi Jewish ancestry and high adjusted PMD (Table 2). The OR for a woman with the most Ashkenazi Jewish ancestry, as defined by PCA, having high adjusted PMD versus low adjusted PMD remained approximately 2. We adjusted for two additional variables outside the model shown in Table 2: breast volume and family history of breast cancer in a first-degree relative. Adjusting for the association between PC1 and breast volume moderately increased the significance of the association between PC1 and adjusted PMD (OR = 2.26, 95% CI = 1.41 to 3.62, *P *= 0.001). Adjusting for family history of breast cancer did not attenuate the association (OR = 1.98, 95% CI = 1.25 to 3.14, *P *= 0.004).

We performed additional analyses on individuals whose genetics suggested partial Ashkenazi Jewish ancestry to determine whether partial Ashkenazi Jewish ancestry was also associated with increased adjusted PMD. We examined the individuals who fell between Group 1 (Northern European ancestry) and Group 4 (Ashkenazi Jewish ancestry) on PC1 (Figure 1). The second PC with the ancestral populations included divided this middle group into individuals who clustered with known Southern European groups (Group 2) and individuals who did not (Group 3). The IBD pattern in individuals in Group 3 supported our hypothesis that this group reflected an admixture between people of Ashkenazi Jewish ancestry and European ancestry, with Group 3 having higher within-group IBD than Group 2 as well as higher between-group IBD with Group 4 than did Group 2 (Table S6 in Additional File 1).

We compared the probability of having high adjusted PMD between the group with mixed Ashkenazi Jewish ancestry (Group 3) and the group with Southern European ancestry (Group 2). We adjusted this analysis for PC1 since women in Group 3 had slightly lower values on PC1 than did Group 2 (-0.02 vs. -0.01; *P *= 0.0007). We found significantly higher probability of high adjusted PMD among Group 3 compared with Group 2 (OR = 2.10; 95% CI = 1.05 to 4.21). This finding suggests that having partial Ashkenazi Jewish ancestry may contribute to an increased risk of having high adjusted PMD.

## Discussion

We performed an analysis of genetic ancestry and age-adjusted and BMI-adjusted PMD, a strong risk factor for breast cancer. We found that the highest value of Ashkenazi Jewish ancestry, as identified by PCA, was associated with a twofold greater risk of having an adjusted PMD in the top quintile. When we analyzed women by clusters of ancestry, women who clustered with Ashkenazi Jews had a 1.6-fold greater likelihood of having higher adjusted PMD compared with women who clustered with Northern Europeans. This association was independent of total breast volume, parity, menopausal status, and postmenopausal hormone therapy. In addition, women who are likely to have partial Ashkenazi Jewish ancestry by PCA and IBD analysis also had higher adjusted PMD.

The identification of an ethnic group with higher adjusted PMD has significant implications for strategies to identify the genetic basis of this trait. Ashkenazi Jews have probably undergone a population bottleneck followed by rapid expansion, consistent with being a founder population [41,42]. Founder populations are more likely to have unique variants that are otherwise absent or exceptionally rare in other populations [43-46]. Since a genome-wide association study has only identified variants that account for <1% of the variance in adjusted PMD [30], but adjusted PMD is estimated to be approximately 60% heritable [24-27], the vast majority of heritability for adjusted PMD remains unexplained. Our finding suggests that women of Ashkenazi Jewish ancestry may have unique genetic variant(s) or higher frequencies of variants that predispose to higher adjusted PMD.

Although a genetic effect is a plausible explanation for the higher adjusted PMD in Ashkenazi Jewish women, we cannot rule out unmeasured nongenetic confounders. We adjusted for some factors known to be associated with PMD including age at parity, menopausal status, and use of postmenopausal hormone therapy. However, we did not adjust for other factors such as age at menarche or number of children. The finding that women of partial Ashkenazi Jewish ancestry also have higher adjusted PMD supports a genetic basis for the increased adjusted PMD in Ashkenazi Jews, although it is also possible that women of mixed Ashkenazi Jewish descent are exposed to the same environmental factors as women of Ashkenazi Jewish descent.

One limitation of our study is that we did not have information about whether these women self-identified as Ashkenazi Jews. However, other genetic studies with self-identification information have identified Ashkenazi Jews as a cluster among US Caucasians [47,48]. Furthermore, individuals who self-identify as having partial Ashkenazi Jewish ancestry can also been identified by PCA [49].

Another limitation of our study was that our analysis depended on both the measurement of PMD using the SXA approach and on a sampling scheme that sampled the top and bottom quintiles. The SXA measurement of PMD is known to have high reproducibility [6] and has been associated with breast cancer risk [5]. In addition, we found an association between high adjusted PMD and family history of breast cancer, as has previously been observed with qualitative measures of breast density [50,51]. However, it is possible that the association between adjusted PMD and genetic ancestry is only apparent when measuring PMD using SXA and is an artifact of that method; additional studies of PMD and ancestry will be necessary to confirm that the association remains when different methods are used to measure PMD. In addition, our analysis sampled the top and bottom quintiles of age-adjusted and BMI-adjusted PMD. The association between genetic ancestry and this trait may be due to a differential effect of age or BMI on PMD in Ashkenazi Jews compared with other Caucasians. We calculated the BMI using self-reported height and weight, which can underestimate high BMI values and overestimate low BMI values [52].

Finally, our adjustment for the effect of BMI on PMD as part of the sampling did not completely eliminate the association between PMD and BMI. We noted an association between BMI and adjusted PMD, even after we had adjusted PMD for BMI. We believe this association was due to a nonlinear relationship between BMI and PMD, especially at higher BMIs. We therefore stratified by BMI quartile and by decile and re-adjusted for BMI in the multivariate analysis using these categories, and did not detect any attenuation of the main association between ancestry and adjusted PMD. Future studies of PMD may benefit from adjusting for BMI initially by categories rather than using a linear regression to avoid having to adjust twice.

The Ashkenazi Jewish population has been reported to have higher rates of breast cancer compared with other Caucasian populations [53], which may be at least partially explained by its high prevalence of two founder mutations in BRCA1 and one founder mutation in BRCA2 [54]. However, the increased prevalence of BRCA1 and BRCA2 germline mutation carriers is unlikely to explain the association of adjusted PMD with Ashkenazi Jewish ancestry, as two studies have demonstrated no association between BRCA1 or BRCA2 mutation status and PMD [55,56]. Based on our data we cannot determine whether increased PMD in Ashkenazi Jewish women is associated with an increased risk of breast cancer independent of BRCA1 and BRCA2. Ashkenazi Jews may have higher PMD because of genetic or environmental factors that increase PMD but have no impact on breast cancer risk. Alternatively, higher PMD in Ashkenazi Jews may result from previously unknown genetic risk factors for breast cancer development.

## Conclusions

In summary, women of Ashkenazi Jewish ancestry are more likely to have high age-adjusted and BMI-adjusted PMD. Environmental risk factors, genetic variation, or both may explain this finding. Ashkenazi Jews are a founder population with substantially higher IBD compared with other populations. One or more genetic variant(s) unique to this population may therefore increase PMD. Further research is needed to uncover potential genetic determinants underlying the higher adjusted PMD in this group, which in turn may shed new light on the biologic mechanisms of PMD.

## Abbreviations

BMI: body mass index; CI: confidence interval; IBD: identity by descent; OR: odds ratio; PC: principal component; PC1: first principal component; PCA: principal components analysis; PMD: percent mammographic density; SNP: single nucleotide polymorphism; SXA: single X-ray absorptiometry.

## Competing interests

The authors declare that they have no competing interests.

## Authors' contributions

JLC performed the statistical analysis and data interpretation and drafted the manuscript. KK obtained funding, contributed to data acquisition and interpretation of the results, and made critical reviews to the manuscript. JAS performed the PMD measurements and made critical reviews to the manuscript. SRC obtained funding, contributed to data acquisition and made critical reviews to the manuscript. DH and SH made contributions to the bioinformatics analysis and made critical reviews to the manuscript. EZ designed the study, obtained funding, provided interpretation of the data and helped to draft the manuscript. All authors read and approved the manuscript for publication.

## Supplementary Material

Additional file 1 **Table S1 describes the number of samples excluded from the analysis and the reasons they were excluded**. **Table S2 **describes the number of SNPs excluded from the analysis and the reasons they were excluded. **Table S3 **includes the analysis of association between PC1 and PMD by quartiles and deciles of BMI. **Table S4 **demonstrates the association between PMD and population subgroups (Groups 1 to 4) rather than using continuous PCs as predictors. **Table S5 **lists the *P *values for associations between PMD and the top ten PCs. **Table S6 **demonstrates the pairwise average IBD segment sharing between pairs of women from different subgroups. **Figure S1 **demonstrates the distribution of PMD before and after transformations and the thresholds for sampling top and bottom quintiles. **Figure S2 **demonstrates the distribution of genetic ancestry of samples from our study in comparison with populations with ancestry from Africa, Europe, East Asia, and America. **Figure S3 **demonstrates the distribution of PMD in relation to BMI. Click here for file
