# Supplementary material for: High mammographic density in women of Ashkenazi Jewish descent
Source: Breast Cancer Res. 2013 May 13;15(3):R40. doi: 10.1186/bcr3424 (PMC4053164; doi:10.1186/bcr3424)
Supplement: Additional file 1 — Table S1 describes the number of samples excluded from the analysis and the reasons they were excluded. Table S2 describes the number of SNPs excluded from the analysis and the reasons they were excluded. Table S3 includes the analysis of association between PC1 and PMD by quartiles and deciles of BMI. Table S4 demonstrates the association between PMD and population subgroups (Groups 1 to 4) rather than using continuous PCs as predictors. Table S5 lists the P values for associations between PMD and the top ten PCs. Table S6 demonstrates the pairwise average IBD segment sharing between pairs of women from different subgroups. Figure S1 demonstrates the distribution of PMD before and after transformations and the thresholds for sampling top and bottom quintiles. Figure S2 demonstrates the distribution of genetic ancestry of samples from our study in comparison with populations with ancestry from Africa, Europe, East Asia, and America. Figure S3 demonstrates the distribution of PMD in relation to BMI. [file bcr3424-S1.DOC]

**Supplementary Information**

**Supplementary Table 1.** Number of samples and reasons excluded.

**Supplementary Table 2.** Number of SNPs and reasons excluded.

**Supplementary Table 3.** Stratification by BMI does not change the association of PC1 and adjusted PMD. In (a) we stratify by BMI quartile and in (b) by BMI decile and calculate the association between PC1 and adjusted PMD in each stratum. Across all quartiles and nine of ten deciles, the association remains positive. The multivariate adjusted OR (adjusting for parity and menopausal status as well as BMI) for the association of PC1 and adjusted PMD when dividing BMI by quartiles is 2.2 (95% CI 1.4-3.6); this value is reported in the main text. The same OR when dividing BMI by deciles is 2.2 (95% CI 1.3-3.6).

|  | **OR of high adjusted PMD at PC1 = 1** |
| --- | --- |
| **BMI Quartile 1** | 4.5 (1.3-16) |
| **BMI Quartile 2** | 1.9 (0.8-4.5) |
| **BMI Quartile 3** | 2.6 (1.1-6.2) |
| **BMI Quartile 4** | 1.8 (0.7-4.8) |

(a)

|  | **OR of high adjusted PMD at PC1 = 1** |
| --- | --- |
| **BMI Decile 1** | 10 (0.5 - 200) |
| **BMI Decile 2** | 4.7 (0.6-34) |
| **BMI Decile 3** | 2.7 (0.6-12) |
| **BMI Decile 4** | 2.4 (0.6-10) |
| **BMI Decile 5** | 1.4 (0.4-5) |
| **BMI Decile 6** | 3.0 (0.8-11.3) |
| **BMI Decile 7** | 3.5 (0.9-14) |
| **BMI Decile 8** | 1.4 (0.3-6.4) |
| **BMI Decile 9** | 0.6 (0.1-4.2) |
| **BMI Decile 10** | 2.4 (0.4-13) |

(b)

**Supplementary Table 4.** Number of women with high vs low adjusted PMD in each genetic ancestry group. Groups 1-4 are defined by the first 2 principal components with ancestral populations included as displayed in Figure 1 of the main text. Odds ratios are of having high adjusted PMD in the group compared to group 1. Multivariate adjustment is for BMI, parity, and menopausal status as in Table 2 of the main text. We note that group 3 (mixed Ashkenazi Jewish descent) has higher adjusted PMD than group 4 (Ashkenazi Jewish descent), but that this difference is not significant and may be due to chance.

|  | **High Adjusted PMD** | **Low Adjusted PMD** | **OR of High Adjusted PMD** | **MV Adjusted OR** |
| --- | --- | --- | --- | --- |
|  | N (%) | N (%) | OR (95% CI) | OR (95% CI) |
| Group 1 (Northern European) | 290 (47) | 330 (53) | -- | -- |
| Group 2 (Southern European) | 60 (50) | 60 (50) | 1.14 (0.77-1.68) | 1.08 (0.71-1.65) |
| Group 3 (Mixed Ashkenazi Jewish) | 37 (69) | 17 (31) | 2.48 (1.37-4.49) | 2.36 (1.26-4.50) |
| Group 4 (Ashkenazi Jewish) | 87 (58) | 62 (42) | 1.60 (1.11-2.29) | 1.77 (1.19-2.61) |

**Supplementary Table 5.** Association of each of the top ten principal components with adjusted PMD. *P*-values are calculated using Wilcoxon rank-sum test.

|  | ***P*-value for association with adjusted PMD** |
| --- | --- |
| **PC1** | 0.004 |
| **PC2** | 0.2 |
| **PC3** | 0.3 |
| **PC4** | 0.7 |
| **PC5** | 0.2 |
| **PC6** | 0.1 |
| **PC7** | 0.03 |
| **PC8** | 0.3 |
| **PC9** | 0.8 |
| **PC10** | 0.3 |

**Supplementary Table 6.** Mean number of centimorgans of shared IBD between each group of Caucasian American individuals as divided by the PC space (Figure 1 in the main text). Groups are divided based on proximity in the PC space to ancestral European populations.

|  | **Group 1 (Northern European)** | **Group 2 (Southern European)** | **Group 3 (Mixed Ashkenazi Jewish)** | **Group 4 (Ashkenazi Jewish)** |
| --- | --- | --- | --- | --- |
| **Group 1 (Northern European)** | 6.0 |  |  |  |
| **Group 2 (Southern European)** | 4.7 | 4.4 |  |  |
| **Group 3 (Mixed Ashkenazi Jewish)** | 5.0 | 4.5 | 9.0 |  |
| **Group 4 (Ashkenazi Jewish)** | 4.0 | 3.9 | 12.7 | 23.2 |

**Supplementary Figure 1.** Distribution of PMD measures across the population of women in the CPMCRI cohort.

(a) Distribution of the average SXA percent density measurement

(b) Distribution of the square root of (a)

(c) Distribution of the residuals of a linear regression model of (b) using BMI and age as variables. Green and red lines show 20^th^ and 80^th^ percentiles outside of which low and high density women were sampled.

**Supplementary Figure 2.** Self-identified Caucasian Californian women (from our data set) cluster with European samples from CEPH diversity data set in PCA. The four Caucasian Californian women that do not cluster tightly with the European samples were excluded from analysis (see Supplementary Table 1).


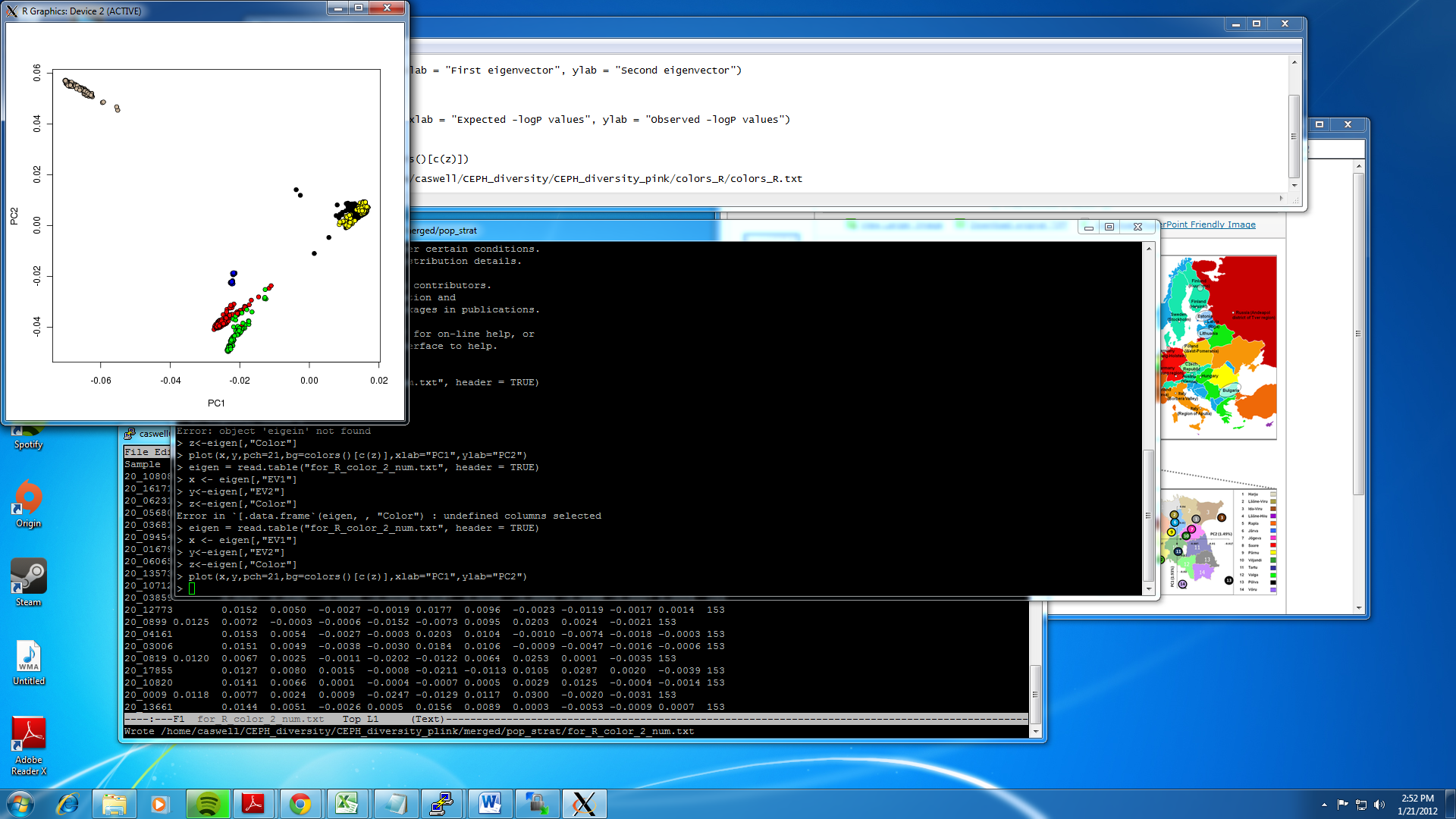


*Black* – Women in our sample

*Yellow* – European

*Tan* – African

*Blue* – Oceanian

*Red* – Asian

*Green* - American

**Supplementary Figure 3.** Relationship between BMI and unadjusted PMD. (a) shows all women in the initial data set. (b) shows the women selected for genotyping based on being in the highest (red) or lowest (blue) quintiles of age- and BMI-adjusted PMD. The excess of women in the highest quintile of age- and BMI-adjusted PMD at higher BMIs seen in (b) results from an assumption of linearity in the non-linear relationship between BMI and unadjusted PMD in (a).

(a)

(b)
